# Use of Hematopoietic Growth Factor in the Management of Hematological Side Effects Associated to Antiviral Treatment for Hcv Hepatitis

**DOI:** 10.4084/MJHID.2010.003

**Published:** 2010-03-31

**Authors:** Paola Mancino, Katia Falasca, Claudio Ucciferri, Eligio Pizzigallo, Jacopo Vecchiet

**Affiliations:** Infectious Disease Clinic – Department of Medicine and Science of Aging. “G. d’Annunzio” University –School of Medicine, Chieti-Pescara – Italy

## Abstract

Haematological abnormalities are common during combination antiviral therapy for chronic hepatitis C. Although dose reduction or discontinuation can easily treat these side effects, they can adversely affect the efficacy of combination antiviral therapy reducing the likelihood of a sustained viral response (SVR). To avoid potentially diminishing a patient’s chance of response, many physicians have begun using growth factors off-label to manage anaemia and neutropenia in hepatitis C. Haematopoietic growth factors are generally well tolerated and they may be useful for managing haematological side effects of anti-HCV therapy improving patients’ quality of life. To date, the role and benefit of these agents during anti-HCV therapy and their positive impact on SVR have not conclusively determined in the published studies. However, the possibility of a benefit to individual outpatients remains, and an individualized approach is recommended. This review explores the incidence, clinical significance, and management of anaemia, neutropenia and thrombocytopenia associated with combination therapy for HCV infection.

## Introduction:

Infection with hepatitis C virus (HCV) is an increasing epidemic with over 180 million people infected worldwide. 1 HCV is the most important cause of chronic liver disease in Italy and is found in nearly 70% of cases either alone or with other factors such as HBV and alcohol abuse. 2 Infection with HCV persists in about 75% of cases and causes various degrees of liver inflammation and fibrosis; in time it may lead to cirrhosis and hepatocellular carcinoma. 3–4

The primary aim of anti-HCV therapy is permanent eradication of the virus or a sustained viral response thereby reducing the risk of progression to end-stage liver disease and improving quality of life. A sustained viral response (SVR) is defined as undetectable plasma HCV-RNA 6 months after the end of treatment, which lasts typically 6–12 months. This leads to a long-term clearance of the virus in 98.3% of patients.^5^ The most effective treatment for chronic HCV infection at present is the combination of pegylated interferon (PEG-IFN) α-2a or α-2b and ribavirin (RBV). Both drugs have a significant effect on virological and histological responses and this combined therapy provides a SVR rate of 40% to 50% in patients with HCV genotype 1 and of 80% in patients with HCV genotypes 2 or 3 in randomized controlled trials.^6^–^7^ In spite of this, at least 50% of patients with genotype 1, which is the most frequent genotype in the Western world (60–90% of those infected), or with other genotypes (e.g. genotype 4) do not respond to this antiviral therapy. The SVR rate decreases dramatically when adherence to treatment is not optimal. Data derived from clinical trials indicate that compliance during treatment and the dose of RBV are important factors for achieving a SVR, particularly among “difficult-to-treat” patients, such as those infected with HCV genotype 1 and those with high baseline levels of HCV-RNA.^8^–^10^ The challenging side-effects of both PEG-IFN and RBV reduce adherence to treatment and also result in high rates of dose reduction and discontinuance of treatment in clinical trials and in practice. Among the side effects of combination therapy, hematologic abnormalities such as anaemia, neutropenia and thrombocytopenia have been reported to result in dose reductions in almost 40% of the subjects^11^ with consequent 10–20% reductions in the virological responses achieved.^6^–^7^

Management of haematological side effects of antiviral therapy for HCV infection can be an important strategy for maximizing treatment outcomes. Many physicians have begun using haematopoietic growth factors “off-label” to manage haematological side effects in patients with chronic hepatitis C. A recent French study reported that 46% of clinicians treating HCV infection used erythropoietic agents and granulocyte colony stimulating factor but there was considerable variation in the molecules and regimen used.^12^ Unfortunately, no official guidelines currently exist, and many issues remain unresolved. This article reviews the haematological adverse effects of antiviral treatment for hepatitis C and the use of haematopoietic agents as potential adjuvant therapy. For this review, a literature search of peer-reviewed articles was made in Medline database for the period between 1991 (FDA approval of epoetin alpha) and 2010.

## Anaemia

### Definition and frequency:

The single most common adverse event of antiviral treatment is anaemia. World Health Organization defines anaemia as haemoglobin levels < 13 g/dL in men and < 12 g/dL in women. During the first 4 weeks of combination treatment, mean haemoglobin levels decrease by an average of 2 to 3 g/dl, with an impaired compensatory reticulocytosis.^6^–^7^,^13^ Haemoglobin concentrations have been reported to decrease to below 12 g/dl (mean decrease 3.7 g/dl) in 52% of patients receiving combination treatment with PEG-IFN α-2a plus RBV.^14^ Significant anaemia (haemoglobin < 10 g/dL) has been observed in up to 9–13% of patients receiving combination therapy with IFN and RBV.^6^ Moderate anaemia (haemoglobin < 11 g/dL) may be seen in 30%.^15^ Female gender, age > 60, higher RBV dose by body weight (12mg/kg or more), the rate of haemoglobin reduction at week two, Asian race and a reduced creatinine clearance are predictors of the development of anaemia with anti-HCV therapy.^16^–^17^

### Pathogenesis:

The aetiology of the anaemia induced by HCV therapy is multifactorial. It is a “mixed anaemia”, in which both haemolysis and bone marrow suppression occur simultaneously.^18^ Clinically, however, the contribution of RBV to anaemia during combination treatment overshadows the effects of IFN on the bone marrow. RBV induces a dose-dependent haemolytic anaemia which is reversible within 4–8 weeks of drug discontinuation. RBV achieves an extensive accumulation in erythrocytes subsequent to active unidirectional transmembraneous transport. Its activation to RBV triphosphate leads to a marked depletion of adenosine triphosphate (ATP). Because erythrocytes lack enzymes to hydrolyze RBV triphosphate, it accumulates in these cells. ATP deficiency impairs the antioxidant defence, allowing oxidative membrane damage to occur. This leads to an accelerated extravascular haemolysis by the reticulo-endothelial system.^13^,^19^

RBV-independent haematologic effects may also occur in HCV patients receiving combination treatment. To a lesser extent, IFN contributes to anaemia by suppressing bone marrow function^20^, limiting erythroid-progenitor-cell proliferation, increasing apoptosis of erythroid cells, promoting autoimmune haemolytic reactions, reducing renal function and impairing compensatory reticulocytosis to RBV-related haemolytic anaemia.^18^,^21^ Hence, the combined actions of these drugs result in a “mixed” anaemia.

Moreover, the anaemia of chronic diseases can be observed in patients with chronic HCV infection. Its pathogenesis is complex, involving impaired iron reutilization, low-grade haemolysis, shortened red blood cell (RBC) lifespan, hyposecretion of erythropoietin (EPO) and tissue hyporesponsiveness to EPO. These effects are thought to result from the actions of inflammatory cytokines,^22^–^25^ which, among other effects, increase the production of hepcidin, an iron regulatory hormone synthesized predominantly in the liver.^26^ Hepcidin induces decrease in plasma iron and sequestration by macrophages resulting in anaemia of chronic disease. Recent reports have shown inadequate hepcidin expression in chronic hepatitis C facilitating the iron deposition in the liver.^27^

A blunted production of EPO complicates the pathophysiology of anaemia. EPO is an endogenous glycoprotein hormone which is the primary regulator of the rate of erythropoiesis.^28^ EPO is regulated at the level of the kidney: when oxygen delivery to the kidney is suboptimal, EPO is secreted and moves to its site of action – the bone marrow – where it enhances erythrocyte proliferation and maturation. It binds to specific receptors on the cell surface of RBC precursors in the bone marrow, stimulating the formation of enucleated reticulocytes, which rapidly mature into RBCs, causing an increase in the circulating red blood cell mass.^29^ Normally, a decrease in the haemoglobin level is accompanied by an increase in the serum EPO level, which will ultimately normalize the haemoglobin level.^30^ The relationship between haemoglobin and EPO is less apparent in patients with chronic diseases, such as cancer and chronic viral infections. HCV-infected patients who were treated with PEG-IFN/RBV appeared to have inappropriately low levels of endogenous EPO for their degree of anaemia.^31^ Although serum levels of endogenous EPO increased during the combination treatment, the haemoglobin level did not return to normal, suggesting that the increase in endogenous EPO was not sufficient to fully compensate for the degree of anaemia.^32^ In addition, even though EPO is synthesized primarily in the kidney, other organs, such as the liver and brain, also produce it. An inadequate EPO production by HCV-infected hepatocytes in the liver also may contribute to a subnormal rise in EPO. Thus the erythropoietic response in HCV-infected patients appears to be decreased.^31^–^32^

### Effect of anaemia on Ribavirin dose and SVR:

RBV-induced anaemia is the main reason for dose reduction and discontinuance,^33^ adversely affecting outcome since it reduces the probability of achieving SVR.^11^ However, maintaining the RBV dose is crucial to the outcome in patients with chronic hepatitis C who are treatment naive or who did not respond to a previous course of therapy.^34^ According to a population pharmacokinetic and pharmacodynamic analysis of RBV in patients infected with hepatitis C, higher concentrations of RBV in whole blood after 4 weeks of treatment were associated with a higher rate of response, and the probability of response increased with increasing concentration.^35^ Clinical data investigating the effects of treatment adherence on SVR in genotype 1 patients found that patients receiving less than 80% of the dose of either PEG-IFN or RBV for less than 80% of the duration of therapy had suboptimal viral response.^8^ Another study showed that genotype 1 patients who received < 60% of cumulative dose of RBV had significantly reduced SVR.^36^ More recently, the cumulative RBV dose to week 12 has been shown to be significantly associated with early viral response (EVR) and SVR in genotype 1 patients.^37^ Similar data was also generated by the IDEAL study, which compared the rate of adverse events, safety profile and SVR of treatment with PEG-IFN α-2a or α-2b plus RBV. Increased exposure to RBV was associated with an increased likelihood of SVR.^38^

Furthermore, anaemia can have negative effects on both cerebral function and quality of life [39] because of it can exacerbate other treatment-related side effects such as dyspnoea and fatigue. So it has marked adverse effects on compliance to the anti-HCV treatment.

## Management Of Anaemia

### Ribavirin dose modification-discontinuation:

The standard-of-care management for patients who develop anaemia during HCV therapy with IFN/RBV is RBV dose reduction by 200 mg/day if haemoglobin levels decreases to < 10 g/dL, and drug discontinuation when haemoglobin levels drop to < 8,5 g/dl. Since such intervention can have adverse implication for SVR rates other alternative approaches are required.

### Erythropoietic growth factors Pharmacology:

To avoid potentially diminishing a patient’s chance of response, many physicians have begun using growth factors such as recombinant human erythropoietin (rHuEPO) off-label to manage anaemia in hepatitis C with the objective of maintaining the RBV dose.^40^ The rHuEPO agents are the biosintetic form of the hormone EPO. Although there are small differences in the sugar profile of the endogenous and recombinant hormones, their action in the human body is identical and all rHuEPO bind and activate the EPO receptor. So their administration stimulates RBC production and predictably increases haemoglobin concentrations; it has also proved to be remarkably well tolerated and highly effective.^41^ However, because of the time required for erythroid progenitors to mature and be released into the circulation, a significant increase in haemoglobin concentration does not usually occur for 2 weeks and may take up to 6 weeks.

There are currently three rHuEPO agents on the market: epoetin alpha (EPO-α), epoetin beta (EPO-β) (in Europe only) and darbepoetin alpha, a structurally different synthetic longer-acting analogue of EPO-α. They are approved for the treatment of anaemia associated with chronic renal failure, cancer, HIV infection and in the surgical setting, to reduce allogenic blood transfusion.^29^,^41^ EPO-α and EPO-β, both produced by Chinese hamster ovary cells, share the same amino acid sequence as endogenous EPO and have the same physiological effects. However, differences in the manufacturing process between the two glycoproteins reflect differences in their carbohydrate moieties,^42^ which determine differences in the pharmacokinetic and pharmacodynamic properties between these agents. In fact, EPO-β has a prolonged half-life following subcutaneous administration and seems to induce a greater absolute reticulocyte response than EPO-α after subcutaneous administration.^43^ They have been extensively studied in chronic renal disease.

### Precautions:

Adverse events related to rHuEPO are rare and consist of hypertension, headache, reaction at injection site, increased numbers of platelets in the blood and an increased risk of thrombosis.^44^ Generally, after a doubling of the initial EPO dose, a further increment of the dose is not advisable because it increases the risk of thrombosis. However, these data mainly originate from rHuEPO employment in cancer patients, who show different characteristics of response and have a greater risk of thrombosis than PEG-IFN/RBV treated patients.^45^ Patients with chronic HCV infection do not have a pro-coagulative profile and need a different rHuEPO treatment duration. Myocardial infarct and cerebrovascular accident are rare complications in non-renal populations and recent studies have not shown HCV treatment patients to be at increased risk.^46^ Moreover, rHuEPO has been shown to enhance platelet reactivity and platelet counts in patients with alcoholic cirrhosis, an interesting feature during antiviral therapy in thrombocytopenic HCV-positive patients with cirrhosis.^47^ The occurrence of pure red cell aplasia, an uncommon disorder associated with the presence of anti-EPO antibodies, was recently reported in a small number of patients with HCV infection and subjects with chronic renal failure treated with EPO-α.^48^–^49^

Before starting rHuEPO therapy it is important to rule out its contraindications. These include uncontrolled hypertension, iron deficiency anaemia and known hypersensivity to mammalian cell-derived products of human albumin.

### Erythropoietic agents during HCV therap

#### EPO-α:

Despite of the agents available EPO-α has been the agent commonly used in anti-HCV treatments, few clinical trials examining its use in this setting have appeared in the peer-reviewed literature.

In an uncontrolled pilot study, 18 hepatitis C patients who developed anemia (haemoglobin < 10 g/dL or decrease of ≥ 2 g/dL from baseline) during antiviral treatment were given EPO-α 40.000 U weekly, and 13 patients also had their RBV dose reduced. Epoetin restored 72% of the prior decrease in haemoglobin concentration.^50^

Dieterich *et al.* randomized 64 HCV-infected patients with anaemia (haemoglobin < 12 g/dL) during combination PEG-IFN/RBV therapy to EPO-α 40,000 U/week s.c. or “standard of care” (SOC) (dose reduction/discontinuation, transfusion). Patients treated with EPO-α had significantly higher haemoglobin levels at week 16 than the SOC group (13.8 g/dL *vs* 11.4 g/dL, p<0.001). reductions in RBV dose at week 4 were significantly lower in the EPO-α group (p<0.001) and, at study end, 83% of treatment group maintained RBV doses of 800 mg/day or more compared with 54% of the SOC group. EPO-α was reported to be well tolerated.^51^

Afdhal *et al*. conducted a randomized placebo-controlled trial in 185 HCV-infected patients on combination therapy who developed anaemia (haemoglobin < 12 g/dL) to investigate the efficacy of EPO-α in maintaining RBV dose, improving quality of life (QOL) and increasing haemoglobin. EPO-α was initiated at 40,000 U/week and titrated to 60,000 U weekly if haemoglobin did not increase by 1 g/dL after 4 weeks. After 8 weeks, 88% of the patients receiving epoetin maintained RBV dose *versus* 60% of patients receiving placebo. RBV dose did not change significantly from randomization to the end of 8 weeks in the EPO-α group, whereas a significant decrease in RBV dose occurred in the placebo group. Mean haemoglobin increased by an average of 2.2 g/dL in the EPO-α group compared with only 0.1 g/dL in the placebo group (p<0.001). In addition, epoetin significantly improved mean QOL scores and was well tolerated.^52^ A post hoc analysis of this clinical trial, however, subsequently reported that QOL scores of anaemic patients in the trial were significantly lower than those of both the general population and patients who had untreated chronic HCV infection, diabetes mellitus and congestive heart failure.^53^

Although the use of EPO-α can reduce the incidence and severity of anaemia induced by PEG-IFN and RBV, data about its effects on SVR are very limited. Only one prospective, randomized, controlled trial has evaluated the effect of EPO-α on virologic response and SVR. Shiffman *et al.* randomized 150 treatment-naïve patients with chronic HCV genotype 1 into 3 treatment groups: (1) PEG-IFN plus weight-based RBV (13.3 mg/kg/day); (2) PEG-IFN plus weight-based RBV plus EPO-α 40,000 U/week; or (3) PEG-IFN plus higher dose weight-based RBV (15.2 mg/kg/day) plus EPO-α. Epoetin was initiated at the onset of therapy to maintain the haemoglobin between 12 and 15 g/dL and RBV was reduced in decrements of 200 mg if required. The incidence of RBV dose reduction was significantly reduced (p<0.05) and the mean dose of RBV received by these patients was significantly higher. Declines in haemoglobin to less than 10 g/dL were significantly less frequent in group 2 than group 1 (10% *vs* 40%, p<0.05). SVR was similar in group 1 and 2 (19% to 29%) whereas it was significantly greater in group 3 patients (49%, p<0.05) who received a higher starting dose of RBV, mostly by a reduction in relapses. The routine use of EPO-α in all patients at the start of anti-HCV treatment does not appear to be beneficial and failed to enhance SVR given the same starting dose of PEG-IFN and RBV. However a higher dose of RBV at initiation of treatment increases SVR by reducing relapse rates.^54^

#### Darbepoetin-α:

Darbepoetin is a hyperglycosilated protein, which gives it a threefold longer serum half-life, higher in vivo potency, and less-frequent dosing compared with EPO-α, approved for treatment of anaemia associated with chronic renal failure and cancer chemotherapy.^55^

A recent open-label, phase II trial was conducted using darbepoetin-α in 101 patients with chronic hepatitis C. Patients received PEG-IFN and a weight-based dose of RBV (800–1400 mg/day) and patients who developed significant anaemia (haemoglobin < 10.5 g/dL) received darbepoetin-α 3 μg/kg once every 2 weeks; the dose was titrated to achieve a haemoglobin level of 12.0 g/dL. After 81 days of darbepoetin-α, haemoglobin significantly increased by 1.9 ± 1.0 g/dL to 12.1 ± 1.1 g/dL (p<0.0001).^56^

#### EPO-β:

There are limited data about the use of EPO-β for anaemia of chronic HCV patients receiving combination therapy. In a retrospective cohort study, 55 chronic HCV patients who developed anaemia (haemoglobin < 10 g/dL) and symptoms during antiviral treatment were given EPO-β 2000 U per visit, and 33 patients also had their RBV dose reduced. A higher percentage of patients with RBV maintenance was observed in the EPO-β group (71%) compared with untreated group. The mean haemoglobin change from week 12 to week 20 was higher in the EPO-β group than in untreated group, especially for patients receiving a total EPO-β dose of more than 16,000 U (+0.70 g/dL *vs* −0.32 g/dL, P=0.023) and of 10,000–14,000 U (+0.60 g/dL *vs* −0.32 g/dL, p=0.023). Therefore, low dose of EPO-β could maintain RBV dose in certain anaemic HCV-infected patient receiving combination therapy.^57^

Falasca *et al.* randomized 42 HCV-infected patients on combination therapy who developed anaemia (haemoglobin decrease of ≥ 2.5 g/dL from baseline) to EPO-β 30,000 U/week s.c. or “standard of care” (SOC) (dose reduction/discontinuation), to investigate the effects of epoetin on anaemia and SVR. EPO-β was titrated to 30,000 U twice a week if haemoglobin did not increase by 1 g/dL after 4 weeks. One month after the introduction of EPO-β, haemoglobin was increased by about 1 g/dl (p=0.001) in treated group. The viral response at the end of treatment was 95.4% in EPO-β group and 80% in SOC group (p=0.2). SVR was statistically higher in EPO-β group than in SOC group (81.8% *vs* 45%, p=0.03). The administration of EPO-β in HCV-infected patients receiving combination PEG-IFN/RBV treatment increases SVR rates among patients developing anaemia by means of the delivery of optimal dosages of RBV and decreased discontinuance rates for adverse effects.^58^

Whereas few studies have used EPO-β, most practitioners use EPO-β because of its pharmacokinetic and pharmacodynamic properties and efficacy. In a French retrospective study on the use of growth factors in chronic hepatitis C treatment, EPO-β was the main rHuEPO molecule prescribed in a mean dose of 30.000 U weekly (range 2.000–80.000 U).^12^ Recently, an anecdotal report has shown that the use of a high dose of EPO-β in an HCV-infected patient with severe RBV-induced anaemia was well-tolerated and allowed PEG-IFN/RBV treatment to be completed, limiting its adverse effects and an optimal viral response to be achieved.^59^

Summarizing, erythropoietic agents appear to be effective in increasing haemoglobin level, allowing maintenance of the RBV dose and improving the quality of life for the period of time they are used. However, their effects on early and sustained viral response remain uncertain, as do the duration of treatment, optimal dose, and reference level of haemoglobin. Identifying patients with pre-existing anaemia and those at high risk of developing anaemia during anti-HCV treatment facilitates the individualisation of PEG-IFN/RBV therapy with lower RBV dosing or earlier growth factor support.

Possible indications for therapy with rHuEPO include a fall in haemoglobin level by > 4 g/dL, haemoglobin levels of < 11 g/dL, and patients developing symptoms and sign of anaemia (palpitations, dyspnoea, easy fatigability, pallor).^52^ If limited hematotoxicity persists despite reducing the RBV dose to 10.6 mg/kg/day (the minimum effective dose),^6^ initiation of an erythropoietic growth factor therapy may be considered. The aim of this treatment should be to maintain an haemoglobin level of > 11 g/dL and not the return to pre-treatment levels.^60^ The first evidence of a response to rHuEPO administration is an increase in the reticulocyte counts within ten days. If no response occurs in 6–8 weeks despite an appropriate increase in the dose, therapy with erythropoietic growth factors should be discontinued.^60^ If the rate of increase of haemoglobin content with erythropoietic agents therapy is > 1 g/dL over two weeks, rHuEPO dose should be decreased because of association with increased risk of thromboembolic phenomena. Once an adequate haemoglobin level (between 10–12 g/dL) is achieved, RBV dose can be increased to the optimum level. Once started, adjunct rHuEPO therapy may be required until the end of treatment.

Disadvantages of these agents are that they adds another parenteral drug to the patient’s treatment regimen, thereby increasing the costs, inconvenience and potential side-effects. The rHuEPO agents are costly, but when compared with standard care, their use has recently been shown to be cost-effective in managing HCV, by increasing therapeutic compliance, improving the quality of life and avoiding the complications of chronic liver disease.^61^

## Neutropenia

### Definition and frequency:

Interferon therapy is associated with a reduction in peripheral white blood cell counts, both neutrophils and lymphocytes. Neutropenia is defined as a peripheral absolute neutrophil count below 1.0 x 10^9^/L. Similar to haemoglobin levels, neutrophil counts decline rapidly within the first 2 weeks of therapy, stabilize for the duration of therapy, and rapidly return to baseline levels after treatment discontinuation. PEG-IFN result in a greater degree of neutropenia than does non pegylated IFN. With typical doses of PEG-IFN used in HCV therapy the absolute neutrophil count often decreases by 30–50% from baseline. Neutropenia-related dose reductions took place in 24% and 18% of patients receiving PEG-IFN α-2a and α-2b, respectively.^11^,^62^–^63^

### Pathogenesis:

Therapy-related neutropenia is primarily caused by IFN through direct toxicity to the bone marrow. Granulocyte colony stimulating factor (G-CSF) is a cytokine produced by monocytes, macrophages, endothelial cells, and fibroblasts in response to such agents as endotoxin, TNF, interleukin (IL)-1, GM-CSF, IL-3, IL-4 and IFN-γ. G-CSF maintains basal neutrophil counts and generates neutrophilia in response to infections.^64^ A negative feedback effect on G-CSF may result from long-term IFN use.^62^ In the other hand, defective synthesis of endogenous G-CSF during combination treatment may contribute to neutropenia.^65^

### Effects of neutropenia:

Neutropenia is a common reason for dose modification or discontinuation of PEG-IFN [6–7]. Usually, dose adjustments effectively treat this haematologic side effects and less than 1% of patients required permanent drug discontinuation. But the resulting suboptimal dosing and potential impact on virologic response are major concerns. Reducing the dose of PEG-IFN can, like RBV dose reduction, also reduce the likelihood of SVR, although this impact has been less clearly established.

In the large multicenter study of PEG-IFN α-2b and RBV, patients who were randomized to PEG-IFN 1.5 mg/Kg/week for 1 month followed by 0.5 mg/kg/week had significantly lower SVR rates than did those who received 1.5 mg/kg/week for the duration of therapy.^6^ This suggests that maintenance of the optimal dose of PEG-IFN for the entire duration of treatment may also be a determinant of long-term virologic response.

Furthermore, the clinical implications of neutropenia are less clear than for anaemia. Although neutrophil counts can fall to levels that have been associated with an increased risk of bacterial infections in oncology patients, severe infections are uncommon in patients with HCV.

In a retrospective cohort analysis factors associated with neutropenia and its clinical effects during anti-HCV therapy were investigated in 119 patients. Neutrophil counts changed by an average of 34% and stabilized after fourth week of therapy; however IFN was not reduced due to neutropenia. Bacterial infections developed in 18% of patients none of which were neutropenic. The SVR was similar in patients who developed significant neutropenia compared with those who did not (45% *vs* 42%).^66^

In another retrospective study, investigators assessed the severity and rate of infection in 209 patients treated with PEG-IFN plus RBV for 24 or 48 weeks depending on their genotype. Forty-six percent of patients developed neutropenia with absolute neutrophil count < 1500/μL, but reported no increased risk of infection.^67^

These findings suggest that neutropenia may be well tolerated by HCV-infected patients receiving combination therapy. This might be explained by a temporary enhanced innate immune cells activity. A recent study demonstrated that in patients with chronic hepatitis C neutrophil chemotaxis and oxidative burst significantly increased during treatment and returned to baseline at the end of therapy.^68^

## Management Of Neutropenia

### Interferon dose modification-discontinuation:

Unfortunately, guidelines for adjustment of IFN relating to neutropenia are unclear with three large studies failing to suggest a specific absolute neutrophil count values.^6^–^7^,^9^ The package insert of both PEG-IFN α-2a and α-2b recommend dose reduction for patients with neutrophil counts less than 750 cells/mm^3^ and drug discontinuation for those with counts less than 500 cells/mm^3^. The minimum effective dose of PEG-IFN appears to be 1 μg/kg/week. However, the maintenance of the optimal dose of PEG-IFN for the entire duration of treatment may also be a determinant of long-term virologic response. The neutrophil count threshold used for dose modification was extrapolated from data in cancer patients who developed neutropenia related to chemotherapy. The implications of these data for interferon-related neutropenia in patients with chronic hepatitis C are not wholly clear.

### Granulocyte colony-stimulating factor (G-CSF). Pharmacology:

G-CSF is a highly purified, not glycosylated protein produced by recombinant technology in a lab strain of *E. coli* by the addition of a gene expressing the human G-CSF. It induces neutrophil production, differentiation and release from the bone marrow.^69^ Filgrastim, lenograstim and nartograstim are the recombinant version of G-CSF commercially available. They are approved for use in chemotherapy-induced neutropenia and used off-label in HCV therapy. Significant increases in the neutrophil counts can be observed within 24 h of G-CSF administration. G-CSF also appears to cause selected-end-cell functional activation including enhanced phagocytic ability.70 Neutrophil levels usually normalize within 1–7 days (average four days).

### Precautions:

In general G-CSF is well tolerated. Common side effects include mild-to-moderate bone pain secondary to proliferation of neutrophils in the bone marrow. Muscle aches, fever, nausea, vomiting and local skin reactions may also occur. The frequency of bone/muscle pain can be reduced by giving G-CSF either two days before or two days after IFN injection.^71^ Rarely, splenomegaly and spontaneous splenic rupture have also been reported with G-CSF use.^72^–^73^ Marked granulocytosis may occur with prolonged therapy. If neutropenic patients receiving G-CSF develop fever, dyspnoea and lung infiltrates, they should be evaluated for the possibility of adult respiratory distress syndrome that warrant immediate cessation of G-CSF therapy until the resolution of the symptom.

G-CSF is contraindicated in patients with known hypersensitivity to *E. coli*-derived proteins, including filgrastim or any of its components.

### G-CSF during HCV therapy:

Very few studies have investigated the use of G-CSF in patients with chronic hepatitis C.

Van Thiel *et al.* evaluated filgrastim as an adjunct to interferon in 30 HCV-infected patients with advanced liver disease. They were randomly assigned to receive PEG-INF α-2b alone or with 300 microg of filgrastim given twice a week. Although the mean and peak of white blood cell counts were higher for the patients receiving filgrastim, the nadir values were the same between the two treatment groups. Filgrastim was well tolerated and a higher proportion of patients receiving it (53% *vs* 40%) achieved SVR, but this difference was not statistically significant.^74^

A retrospective study of 132 HCV patients receiving PEG-IFN and RBV investigated the benefits of haematological growth factors and G-CSF. 17 patients required filgrastim administration at dose of 30 MU/week. Following G-CSF administration, neutrophil count improved and the optimum PEG-IFN dosage could be obtained or restored in 16 (94%) of the 17 patients treated.^75^

Sharvadze *et al.* studied 47 patients with HCV genotype 1b infection treated with PEG-IFN plus RBV, 41 (87%) of whom developed neutropenia. Neutropenic patients were randomized to filgrastim 300 μg/week s.c. (n=22) or PEG-IFN dose reduction/discontinuation (n=19). In all 22 patients of filgrastim group the drug was well tolerated, normalized neutropenia without modification of interferon dose and improved quality of life.^76^

Koirala *et al.* retrospectively rewieved 163 patients who received combination therapy for HCV. Neutropenia was defined as absolute neutrophil count ≤ 1.0 x 10^9^/L and 30 patients was treated with G-CSF and matched with a group who received anti-HCV treatment without developing neutropenia. G-CSF was given weekly at a dose of 300 μg giving an average rise in absolute neutrophil count of 3.9 x 10^9^/L. Eight patients required dose escalation, two required dose reduction and one stopped due to side effects (rash). There was no statistically significant difference in the SVR between the two groups (61% *vs* 76%, p=0.18).^71^

In the study of Younossi *et al.* conducted in 101 patients with chronic hepatitis C, 38% developed neutropenia with an absolute neutrophil count ≤ 0.75 x 10^9^/L and was treated with filgrastim. After G-CSF was initiated, patients’ absolute neutrophil count increased from 0.75 ± 0.16 x 10^9^/L to 8.28 ± 5.67 x 10^9^/L (p<0.0001). Adverse events of filgrastim were predominantly bone pain and body aches which occurred in 29% of patients treated and splenomegaly was observed in two patients. Finally, treatment with growth factors was independently associated with a SVR and 55% (21 of 38) of patients receiving filgrastim therapy achieved SVR *vs* 40% (25 of 63) who did not receive filgrastim therapy (p=0.07).^56^

In a small case series 8 patients with chronic HCV-related hepatitis who developed neutropenia during antiviral treatment received lenograstim at the dosage of 263 μg weekly. All patients receiving G-CSF completed the antiviral treatment with standard dose of PEG-IFN, with 75% of patients (6 of 8) showing a SVR.^77^

In a recent retrospective, cross-matched study, Koskinas *et coll.*, 232 genotype-1 HCV-infected patients were examined. 19 patients who developed significant neutropenia (neutrophils < 800/mm^3^) was treated with a flexibile scheme of 150–300 μg of G-CSF twice a week and matched with 19 neutropenic patients treated with IFN dose reduction/discontinuation as control group. The mean decline of the neutrophils was similar in two groups and nadir neutrophil values were also not statistically different. A SVR was observed in 32% of patients treated with G-CSF versus 21% of patients in control group. No side effects related to G-CSF was observed.^78^

Overall, reasonable result have been obtained when G-CSF has been utilized in some studies to avoid IFN dose reductions. G-CSF therapy may be considered if neutrophil counts remain < 0.5 x 10^9^/L despite reducing the PEG-IFN dose to the minimum effective level. A suggested dose regimen is starting G-CSF therapy at a dose of 300 μg subcutaneously once weekly, and then adjusting the dose as per the response/requirement. The aim should be to maintain a neutrophil count of ≥ 1000 cells/μL (return to the pre-treatment levels is not the aim). Most patients respond adequately to a G-CSF dose of 300 μg/week, but some patients may require up to 480 μg filgrastim thrice weekly, whereas others may only need 150 μg once weekly. Complete blood counts should be taken twice or thrice weekly to judge the response to therapy. Once an adequate neutrophil count is achieved, IFN dose can be increased to the optimal level. Once started, adjunct G-CSF therapy may be required until the end of the treatment.

## Thrombocytopenia

### Definition and frequency:

A decrease in platelet count may be observed in 10–15 % of patients who are receiving interferon. Thrombocytopenia is defined as a platelet count of 20,000 to < 70,000/mm^3^ and it is a possible cause of interferon dose reductions in less than 4% of patients on combination therapy.^6^ It is more severe with PEG-IFN/RBV therapy as compared to non-pegylated IFN/RBV therapy. It is worst with PEG-IFN monotherapy,^7^ suggesting that use of RBV may blunt the thrombocytopenic effect of interferon as a result of reactive thrombocytosis.

With PEG-IFN, the platelet cont decreases gradually over 8 week, stabilizing thereafter and returning to baseline values within 4 weeks of stopping therapy. This fall is clinical insignificant in most cases and bleeding complication are uncommon.^6^–^7^

In randomized clinical trials of the PEG-IFN, the rate of dose reduction attributed to thrombocytopenia ranged from 3% to 6%.^6^–^7^ However thrombocytopenia associated with chronic liver disease may prevent initiation of treatment and most patients in clinical trials are carefully selected, excluding patients with more advanced liver disease.

### Pathogenesis:

Since thrombocytopenia has an heterogeneous nature several are the possible cause. During anti-HCV combination therapy it is caused primarily by a reversible bone marrow suppression due to effect of interferon. However, others possible mechanisms may be include. Decreased platelet production occurs, due to decreased hepatic production of thrombopoietin (TPO), the major regulator of both megakaryopoiesis and platelet production in human body,^79^ and virus-induced bone-marrow suppression.^80^ In the other hand, an increased peripheral destruction of platelets may coexist, both immune-mediated^81^ and due to portal hypertension and hypersplenism leading to increased splenic platelet sequestration.^82^ HCV binding to platelet membrane with consequent binding of anti-HCV antibody and phagocytosis of platelets, and derangement of host immune system triggering the production of auto-antibodies against platelets glycoproteins are the two most frequently postulated immune mechanisms explaining increased peripheral destruction in HCV-infected cases.^80^–^81^,^83^

## Management of Thrombocytopenia

### Current recommendations:

The usual protocol is to continue with IFN therapy but reduce its dose if the platelet count fails to < 30 x 10^9^/L, and discontinue if it falls to < 20 x 10^9^/L.^60^ G-CSF therapy may be considered if platelet counts remain < 30 x 10^9^/L in spite of reducing PEG-IFN dose to the minimal effective level.

Actually, the anti-HCV combination therapy to eradicate the infection is the most practical strategy in treating HCV-related thrombocytopenia.

### A role for thrombopoietic growth factors?

After the cessation of clinical trials of first generation thrombocytopenic growth factors due to immunogenicity issues, the introduction of non-immunogenic second-generation thrombopoietin mimetics has opened a novel way to treat thrombocytopenia.

Recently, an oral thrombopoietin-receptor agonist, eltrombopag (SB-497115) has been shown to stimulate megakaryocyte proliferation and differentiation and to cause dose-dependent increases in platelet counts in chimpanzees and humans.^84^–^86^ It appears that eltrombopag binds the TPO receptor at a distance from the binding site for TPO and appears to initiate signal transduction by a mechanism different from thrombocytopenic growth factors of first generation.^87^ Thus, eltrombopag has an additive, and not competitive, effect on platelet production. It produces a dose-dependent increase in platelet proliferation and differentiation in chronic immune thrombocytopenic purpura^88^ with no rebound thrombocytopenia following discontinuation treatment.^85^

In a phase II study, eltrombopag has been shown to increase platelet counts in HCV-related cirrhosis. 74 patients with platelet counts of 20–70 x 10^3^/mm^3^ were randomly assigned to receive eltrombopag (30, 50 or 75 mg daily) or placebo daily for 4 weeks. At week 4, none of the controls had an increase in platelets compared with 75–95% of those receiving eltrombopag doses of 30–75 mg respectively. Whereas only 6% of patients in the placebo group completed the 12 weeks antiviral course, the same was completed by 36%, 53% and 65% of patients receiving 30, 50 and 75 mg of eltrombopag respectively.^89^ Although the study was small and thus underpowered, the most commonly reported side effects (headache, dry mouth, abdominal pain, and nausea) were of insufficient severity to require discontinuation of the drug.

Eltrombopag appears to be an adequate candidate for the management of HCV-related thrombocytopenia. Despite the encouraging initial results, further confirmation of the therapeutic efficacy and safety of eltrombopag in phase III trials involving standard-duration courses of PEG-IFN and RBV is required.

## Conclusions:

Haematological abnormalities are common during combination antiviral therapy for chronic hepatitis C. Although dose reduction or discontinuation can easily treat these side effects, they can adversely affect the efficacy of combination antiviral therapy reducing the likelihood of an SVR. Haematopoietic growth factors including rHuEPO and G-CSF are generally well tolerated and they may be useful for managing haematological side effects of anti-HCV therapy improving patients’ quality of life. However, their positive impact on SVR is not substantiated by studies to date. Eltrombopag may allow anti-HCV treatment in patients with thrombocytopenia although further data are required to validate its true indications, dosage, therapeutic efficacy and safety profile. More studies are needed to establish the right dosage schedule and cost-effectiveness of therapy with haematopoietic growth factors in course of combination antiviral therapy.
